# Structural insights into pro-aggregation effects of *C. elegans* CRAM-1 and its human ortholog SERF2

**DOI:** 10.1038/s41598-018-33143-1

**Published:** 2018-10-05

**Authors:** Meenakshisundaram Balasubramaniam, Srinivas Ayyadevara, Robert J. Shmookler Reis

**Affiliations:** 1 McClellan Veterans Medical Center, Central Arkansas Veterans Healthcare Service, Little Rock, AR 72205 USA; 2 Department of Geriatrics, Reynolds Institute on Aging, University of Arkansas for Medical Sciences, Little Rock, AR 72205 USA

## Abstract

Toxic protein aggregates are key features of progressive neurodegenerative diseases. In addition to “seed” proteins diagnostic for each neuropathy (e.g., Aβ_1–42_ and tau in Alzheimer’s disease), aggregates contain numerous other proteins, many of which are common to aggregates from diverse diseases. We reported that CRAM-1, discovered in insoluble aggregates of *C. elegans* expressing Q40::YFP, blocks proteasomal degradation of ubiquitinated proteins and thus promotes aggregation. We now show that CRAM-1 contains three α-helical segments forming a UBA-like domain, structurally similar to those of mammalian adaptor proteins (e.g. RAD23, SQSTM1/p62) that shuttle ubiquitinated cargos to proteasomes or autophagosomes for degradation. Molecular modeling indicates that CRAM-1, through this UBA-like domain, can form tight complexes with mono- and di-ubiquitin and may thus prevent tagged proteins from interacting with adaptor/shuttle proteins required for degradation. A human ortholog of CRAM-1, SERF2 (also largely disordered), promotes aggregation in SH-SY5Y-APP_Sw_ human neuroblastoma cells, since *SERF2* knockdown protects these cells from amyloid formation. Atomistic molecular-dynamic simulations predict spontaneous unfolding of SERF2, and computational large-scale protein-protein interactions predict its stable binding to ubiquitins. SERF2 is also predicted to bind to most proteins screened at random, although with lower average stability than to ubiquitins, suggesting roles in aggregation initiation and/or progression.

## Introduction

Neurotoxic protein aggregation is a hallmark of neurodegenerative diseases including Alzheimer’s disease (AD), Huntington’s disease (HD), and Amyotrophic Lateral Sclerosis (ALS). Aggregates in these neuropathies contain, in addition to disease-specific proteins that are diagnostic for each condition, many common proteins found in insoluble aggregates from multiple progressive diseases and which may play roles in aggregate growth and/or toxicity^1–4^. Failure of protein homeostasis precedes other evidence of neurotoxicity^5–7^, suggesting that it may contribute causally by allowing persistence of misfolded proteins^8,9^. Inherently disordered proteins are predisposed to misfolding, and diverse post-translational modifications (PTMs) — including oxidation, phosphorylation, and acetylation^10,11^ — can introduce structural instability, exposing hydrophobic regions that may interact with those of other transiently or irreversibly denatured proteins^8,11–14^. Although numerous explanations have been offered for the failure of proteostasis in neurodegenerative diseases, the underlying mechanisms remain elusive.

The canonical pathway for ubiquitin-mediated targeting of misfolded proteins to proteasomes is as follows: E3 ubiquitin ligases recognize misfolded proteins and add an initial ubiquitin moiety. Extension of polyubiquitin chains marks proteins for degradation by proteasomes and/or autophagosomes. Shuttle proteins (e.g. RAD23A) bind polyubiquitin tags via a ubiquitin-binding (UBA) domain and escort their cargos to proteasomes^15,16^. Genetic disruption of RAD23A and/or RPN10, a regulatory/docking subunit of the 26S proteasome, leads to accumulation of ubiquitin-tagged proteins^16,17^.

*Caenorhabditis elegans* transgenic strains that simulate neurodegeneration-associated aggregation have proven invaluable in assessing mechanisms of age-dependent proteostasis failure^1,5,18^. For example, nematode strain AM141 expresses Q40::YFP (approximating the glutamine-array-length threshold for penetrance of Huntington’s disease) in body-wall muscle, leading to age-progressive protein aggregation and paralysis^1,19^. Strain CL4176 [*myo-3*p::Aβ_1–42_] expresses the human amyloid peptide Aβ_1–42_ in body-wall muscle, whereas CL2355 expresses Aβ_1–42_ in all neurons, and both show age-progressive behavioral disruptions. These nematode strains model human amyloidopathies, such as β-amyloid deposition in Alzheimer’s disease, and have provided insights into the composition of aggregates, gene activities that favor or oppose aggregation, and pharmacologic or genetic interventions that are protective^1,20–22^.

We recently reported that knockdown of Cytotoxicity-Related Aggregation Mediator-1 (CRAM-1) protects against aggregation in several *C. elegans* models^1^. In the present work, we extended our characterization of CRAM-1 and its human orthologs through both molecular-biology and computational approaches. We found that CRAM-1 contains a UBA-like domain, structurally similar to that of human RAD23A, which interacts with polyubiquitin and thus could compete with RAD23A for ubiquitin binding. We hypothesized that CRAM-1 and its orthologs, despite considerable sequence divergence, may still conserve key properties such as ubiquitin binding, allowing them to block degradation of ubiquitinated proteins. In support of this conjecture, knockdowns of CRAM-1 and SERF2, its closest human ortholog, appear to promote aggregation by very similar mechanisms.

## Results

### CRAM-1 has a UBA-like domain structurally similar to RAD-23

**Figure 1:**
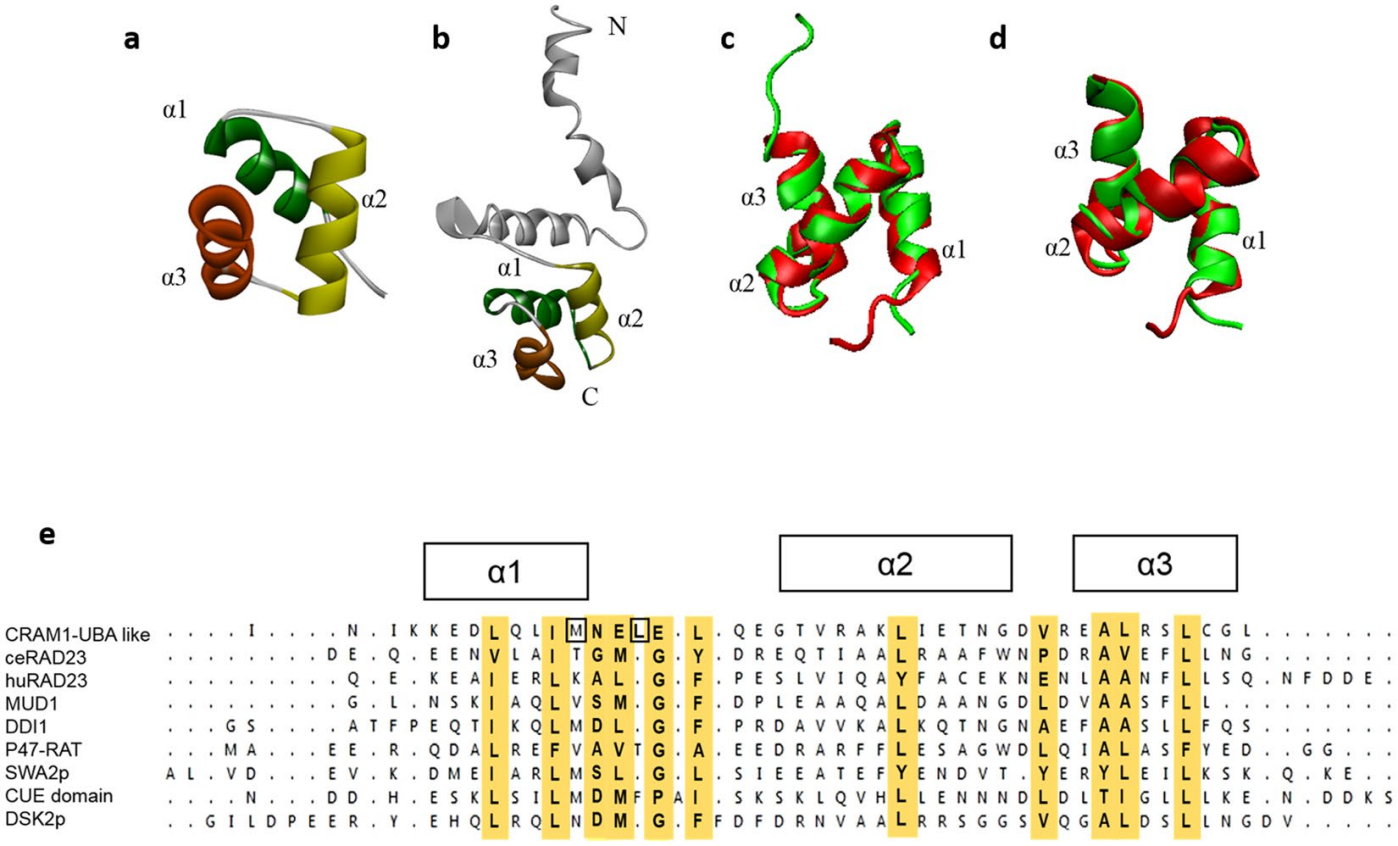
Predicted 3-dimensional structure of CRAM-1 indicates a UBA-like domain. Structures of the CRAM-1 C-terminal region (**a**) and the full-length model (**b**) show the same bundle of three helices connected by loops. (**c**) Structural comparison of the CRAM-1 C-terminal region (red) with the UBA domain of human RAD23A (green). Superimposed structures illustrate structural agreement, with RMSD (root-mean-square deviation) = 1.14 Å. (**d**) C-terminal region of CRAM-1 (red) superimposed on the UBA domain of ceRAD-23 (green), RMSD = 1.02 Å. (**e**) Sequence alignment, based on superimposition of 3-dimensional structures, showing 3 α helices with conserved hydrophobic residues (highlighted in gold) and nearby exposed hydrophobic residues (boxes).

In previous work, we identified CRAM-1 as a minor component of insoluble aggregates from aged adults of *C. elegans* strain AM141 (expressing Q40::YFP in muscle). CRAM-1 is predominantly an unstructured or disordered protein, and preferentially interacts with polyubiquitins^1^. In this work, we further characterize structural features of CRAM-1. First, we modeled its C-terminal region (residues 54 to 96) by an *ab-initio* approach (Fig. 1a). Consistent with the full-length structure (Fig. 1b), which was also predicted by *ab-initio* methods, C-terminal modeling predicted CRAM-1 to have three alpha helices (α1, α2, and α3) of moderate stability, connected by loops. Similar structures are characteristic of UBA-like superfamily domains^23,24^, although full-length CRAM-1 lacks any sequence similarity to other UBA-domain proteins including RAD23, Sequestosome-1/p62, DSK2, and MUD1. UBA-domain proteins show little similarity at the sequence level except for moderately conserved hydrophobic residues, but instead display structural similarity with respect to the three-helix bundle^25^. We assessed structural congruity of the predicted C-terminal region of CRAM-1 with well-established UBA-domain proteins including MUD1 (yeast), RAD23A (human) and SQSTM1/p62 (human) using the Swiss-PDB viewer^26^ and the “multiseq” plugin for VMD^27^. The C-terminal region of CRAM-1 aligns quite closely with the UBA domain (UBA2) of human RAD23A^28^, indicating high structural homology (the two ribbon models are superimposed in Fig. 1c; RMSD ≈ 1.14 Å), but deviates substantially more from the SQSTM1/p62 UBA domain (not shown; RMSD ≈ 3.03 Å).

Since CRAM-1 is a *C. elegans* protein, we modeled the UBA domain of *C. elegans* RAD-23 (hereafter denoted as *ce*RAD-23) and compared it to CRAM-1. The CRAM-1 UBA-like domain showed even closer structural resemblance to *ce*RAD-23 (ribbon models are superimposed in Fig. 1d; RMSD ≈ 1.02 Å) than to human RAD23A. The interactions of UBA-domain proteins with ubiquitins are largely mediated by hydrophobic interactions, involving conserved core residues (chiefly in UBA alpha-helices) supplemented by additional, unstructured UBA-domain residues, interacting with conserved hydrophobic residues in ubiquitin^24,28^. We examined the CRAM-1 UBA-like (C-terminal) region for hydrophobic residues conserved with known UBA proteins. CRAM-1 possesses many of the hydrophobic residues conserved among known UBA-domain proteins including the 3-helix bundles of DSK2, SWA2p, DDI1, MUD1 and RAD23^24^, which are considered crucial for ubiquitin interactions (Fig. 1e, highlighted in gold), and a few additional exposed hydrophobic residues nearby (Fig. 1e, boxes). Together, these findings support the hypothesis of structural conservation between the CRAM-1 UBA-like domain and previously identified UBA domains, including those of human and nematode RAD23.

### Interaction of CRAM-1 with mono- and oligo-ubiquitins

Proteasome-trafficking adaptor proteins, including RAD23A, DSK2, and MUD1, bind ubiquitin chains via UBA domains^29^. We predicted stable interaction of the CRAM-1 UBA-like domain with ubiquitins^1^; we now estimate its docking affinity for mono-, di-, and tetra-ubiquitin (Ub1, Ub2, and Ub4). As positive controls for ubiquitin binding, we modeled docking for MUD1 and RAD23A, previously reported UBA-domain proteins^24,28,30^. Docking simulations predict stable interaction of MUD1 and RAD23A with Ub1 and Ub2 similar to those determined by NMR chemical-shift assays^24,28,30^, supporting the validity of our modeling conditions (Supplementary Fig. 1a,b).

**Figure 2:**
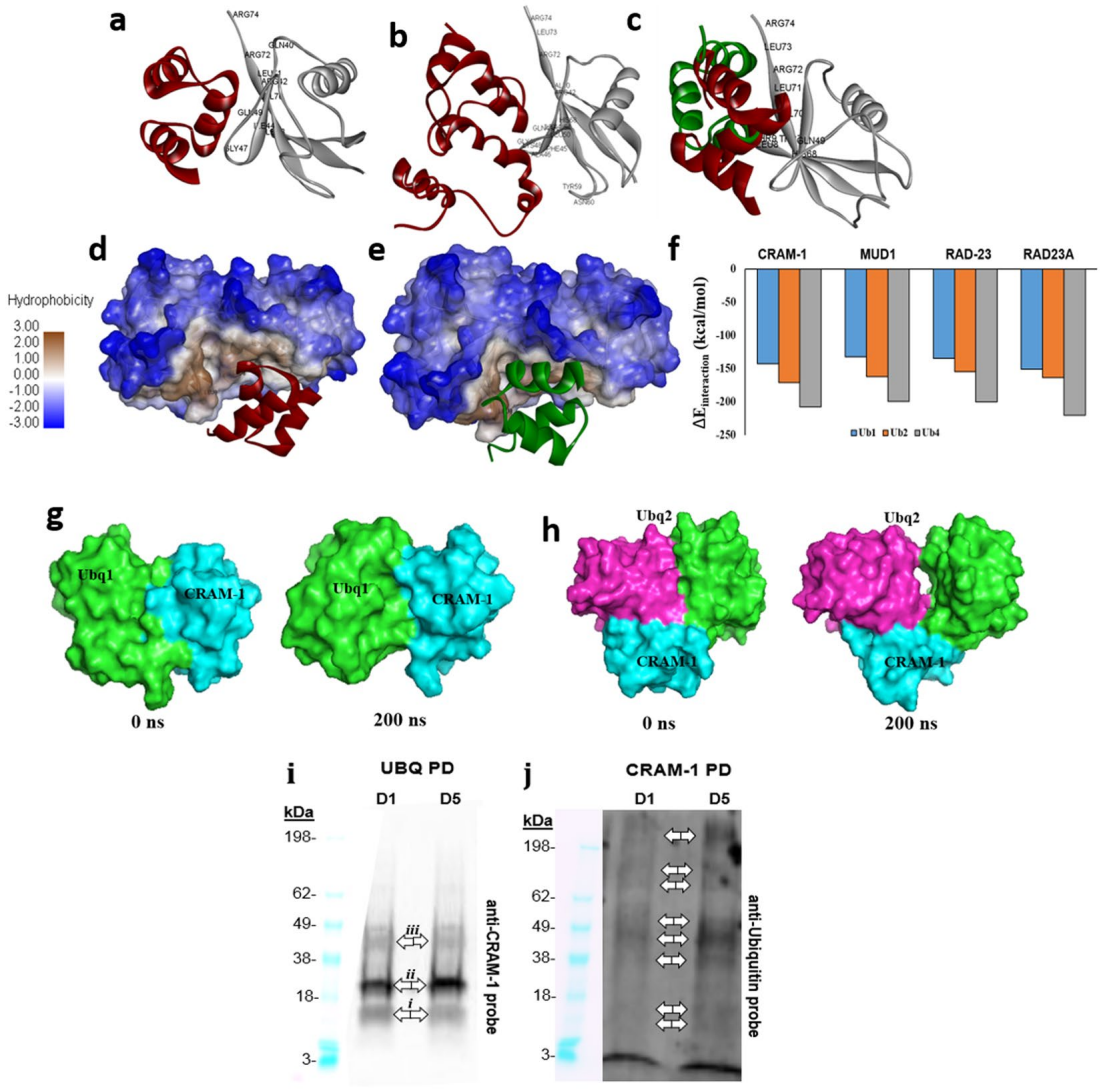
CRAM-1 and RAD-23 have similar interactions with ubiquitins. (**a**) The CRAM-1 UBA-like domain (red) interacts with Ub1 (mono-ubiquitin, gray); interacting amino acids of ubiquitin are labeled. (**b**) The full-length structure of CRAM-1 (red) binds the same region of ubiquitin, contacting most of the same ubiquitin residues that were predicted to interact with the CRAM-1 C-terminus alone. (**c**) Structural superimposition of *ce*RAD-23 (green) and CRAM-1 (red), showing their predicted interactions with mono-ubiquitin via the same ubiquitin aspects. (**d**,**e**) Binding of CRAM-1 (**d**, red) or *ce*RAD-23 (**e**, green) ribbon models to di-ubiquitin (space-filling model) is facilitated by hydrophobic interactions (brown; see scale at left). (**f**) Predicted interaction energies (ΔE_interaction_) for CRAM-1 (UBA-like) and 3 other UBA proteins, each with mono-, di-, and tetra-ubiquitin. (**g**,**h**) Simulated structures of complexes between the CRAM-1 UBA domain (cyan) and either mono-ubiquitin (light green; **g**) or di-ubiquitin (pink and light green; **h**) appear stable over a 200-ns simulation. (**i,j**) Western blot analyses for CRAM-1 interaction to ubiquitin: lysates from wild-type N2 worms at days 1 and 5 were immuno-precipitated with biotinylated CRAM-1 antibody (**i**) or antibody to ubiquitin (**j**), resolved in polyacrylamide gradient gel lanes (10% w/v, BioRad), electroblotted to nylon membranes, and probed with antibody to CRAM-1 (**i**) or ubiquitin (**j**), followed by peroxidase-tagged antibody to IgG and chemiluminescence imaging (Western ECL kit, Bio-Rad).

Using the same parameters, we calculated docking orientations and energies for the CRAM-1 UBA-like domain and *ce*RAD-23 UBA, each interacting separately with Ub1, Ub2 and Ub4. The results (Fig. 2) indicate that interactions of the CRAM-1 UBA-like domain with mono-, di- and tetra-ubiquitins are quite similar to MUD1-ubiquitin interactions (Supplementary Fig. 1). These docking results predict that CRAM-1 and *ce*RAD-23 can bind to the same region, largely via the same residues in ubiquitin (Ub1, Ub2; Fig. 2c–e). It is noteworthy that human RAD23A is predicted to interface with a ubiquitin surface (Supplementary Fig. 1) that is supported by NMR chemical-shift data^28,30^ but differs from the ubiquitin aspects predicted to be bound by *C. elegans* RAD-23, which instead coincide with the interface of yeast MUD1 binding to ubiquitin. For this reason, in subsequent studies we considered only comparisons between proteins that co-evolved — *C. elegans* CRAM-1 with *ce*RAD-23, and human SERF2 with human RAD23A.

RAD23A and MUD1 belong to a subset of UBA-domain proteins that recognize K_48_-linked polyubiquitin chains, and preferentially target their cargos to proteasomes^24,31^. The ubiquitin structures we employed were reported in NMR and X-ray-crystallographic studies of K_48_-linked di- and tetra-ubiquitins, respectively^32,33^. Empirically, UBA:ubiquitin binding affinity increases with ubiquitin chain length^24^, so we asked whether this trend is predicted via computational docking and whether it extends to CRAM-1. UBA or UBA-like domains of MUD1, *ce*RAD-23, RAD23A, and CRAM-1 were docked to Ub1, Ub2 (K_48_-linked open conformer), or Ub4 (K_48_-linked). The predicted free-energy drop on ubiquitin docking (ΔE_interaction_) agrees with published observations, indicating comparable gains in affinity as ubiquitin chain length increases, for established UBA domains (MUD1, *ce*RAD-23, and RAD23A), and for the CRAM-1 UBA-like domain (Fig. 2f). Full-length CRAM-1 is predicted to have even greater binding affinity for each ubiquitin target (−180 to −235 kcal/mol; Supplementary Fig. 1c), than the isolated UBA-like domain alone (−140 to −210 kcal/mol).

We then analyzed the stability of CRAM-1 binding to Ub1 or Ub2 by atomistic molecular-dynamic simulation. The structural integrity (stability) of complexes, comprising the CRAM-1 UBA-like domain bound to mono- or di-ubiquitin, was assessed in 200-ns simulations. Both complexes, CRAM-1/mono-ubiquitin (Fig. 2g) and CRAM-1/di-ubiquitin (Fig. 2h), remained stable throughout the simulations.

To test our computational predictions that CRAM-1 could bind to oligo-ubiquitins, we assessed interactions between CRAM-1 and ubiquitin in wild-type (Bristol-N2) adults at two ages, day 1 (pre-gravid) and day 5 (post-gravid) of adulthood. First, ubiquitinated and ubiquitin-interacting proteins were isolated from lysates of wild-type (Bristol-N2) worms by immuno-pulldown (IP) on magnetic beads coated with antibody to ubiquitin. Bound proteins were eluted, concentrated, electrophoresed, and electroblotted onto nylon membranes, which were then probed with antibody to CRAM-1 (Fig. 2i). Prominent, discrete proteins are seen at SDS-gel mobilities (marked by double arrows) corresponding to native CRAM-1 (band ***i***, ~10.8 kDa), mono-ubiquitinated CRAM-1 (***ii***, ~19.3 kDa), and tetra-ubiquitinated CRAM-1 (***iii***, ~45 kDa). Of these bands, only mono-ubiquitinated CRAM-1 (***ii***) appeared more abundant in older worms.

In parallel, lysates from the same wild-type worms were used to recover CRAM-1 and associated proteins, by IP with biotin-tagged antibody against CRAM-1 and capture on streptavidin-coated magnetic beads. IP-recovered proteins were rinsed, electrophoresed, blotted, and probed with antibody to ubiquitin. CRAM-1 appears to bind a wide variety of ubiquitinated proteins that increase in abundance with age (e.g., double arrows in Fig. 2j) in agreement with previous reports of age-associated aggregation in wild-type *C. elegans*^1,34^ and our observation of CRAM-1 in insoluble protein aggregates from aged worms^1^. Ubiquitinated CRAM-1 must make up a rather small fraction of this signal, since only relatively minor bands are seen at mobilities observed in the preceding IP blot (Fig. 2i).

### CRAM-1 could compete with *ce*RAD-23 for binding to polyubiquitin, impairing substrate delivery to *C. elegans* proteasomes

In previous studies, we found that *cram-1* knockdown (KD) by RNA interference (RNAi) protects *C. elegans* against aggregation and its toxicity, utilizing transgenic strains that form aggregates modeling diverse neurodegenerative diseases^1^. If CRAM-1 functions as a UBA-like shuttle protein, then its knockdown should have been detrimental to the worms. Instead, CRAM-1 RNAi was strikingly protective in each model, and reduced aggregate burden in wild-type worms as well as in neuropathy models^1^. In each protein-aggregation model, integrity of the ubiquitin-proteasome system (UPS) was essential for *cram-1* KD-mediated protection^1^.

**Figure 3:**
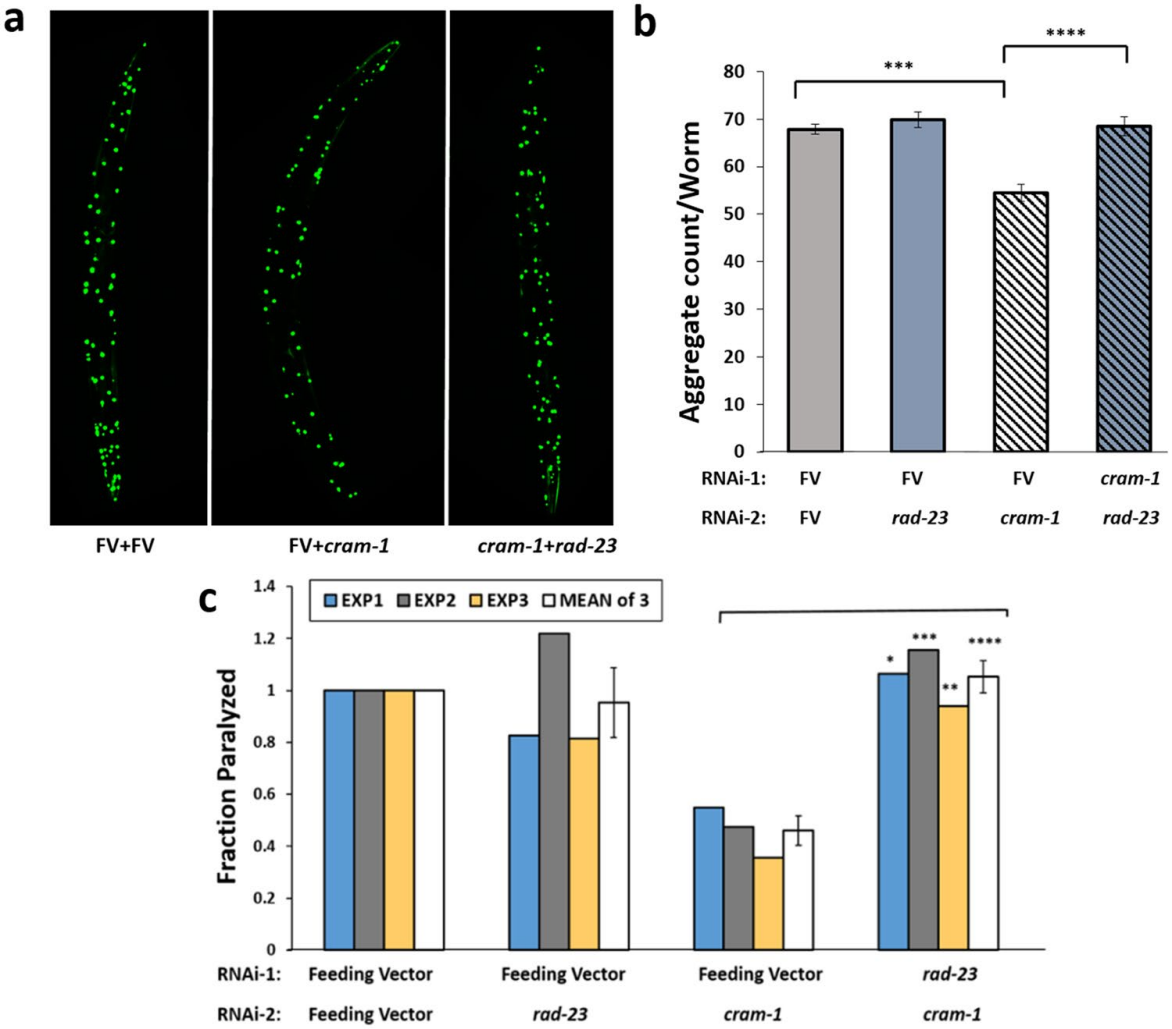
Rescue by *cram-1* knockdown, of both Q40::YFP and Aβ_1–42_ amyloid aggregation, requires RAD23. (**a**,**b**) Protection from Q40::YFP aggregation, conferred by *cram-1* knockdown, is blocked by *rad-23* RNAi. Dual knockdowns were conducted as described. Aggregate numbers per worm, ± SEM, were counted at day 5 post-hatch (D5_PH_) for 10–16 worms per group. Significance (2-tailed *t*-tests): ***P < 3E–5 comparing [FV + FV] to [FV + *cram-1*_*KD*_]; ****P < 10^–4^ comparing [FV + *cram-1*_*KD*_] to [*rad-23*_*KD *_* + cram-1*_*KD*_]. (**c**) CL4176 worms were treated, beginning 36–40 h after egg isolation, with dual RNAi in 3 experiments. *Cram-1* RNAi reduced paralysis relative to [FV + FV] controls, but not when *cram-1*_*KD*_ was paired with *rad23*_*KD*_. Shaded bars show fraction paralyzed, normalized to FV, for 3 independent experiments; open bars summarize combined data ± SEM. Significance by chi^2^ test within each experiment, comparing [*cram-1*_*KD *_ + FV] to [*cram-1*_*KD *_* + rad23*_*KD*_], for 50–100 worms/group: *P < 0.05; **P < 0.01; ***P = 0.001. Treating each experiment as one point per group, ****P < 0.006 by 2-tailed paired *t*-test (white/open bars).

We now predict by computational modeling, and corroborate by immuno-pulldowns, that the CRAM-1 UBA-like domain could interact with ubiquitin and its oligomers, analogous to ubiquitin binding by the *ce*RAD-23 UBA. These combined results led us to hypothesize that, due to structural similarity and conservation of contact residues, CRAM-1 could act as a decoy mimic that competes with *ce*RAD-23 for oligo-ubiquitin binding, but lacks a second recognition domain to convey its cargo to proteasomes or autophagosomes. To test this prediction *in vivo*, we knocked down the expression of *ce*RAD-23 in AM141 worms expressing Q40::YFP in muscle. As in previous experiments^1^, worms were transferred to RNAi plates at 48 h post-hatch, and imaged on day 5 post-hatch (d5_PH_), for quantitation of aggregates. Although *rad-23* knockdown alone had no effect on aggregate counts per worm, relative to controls, *cram-1* knockdown reduced both the number and intensity of aggregates by 23‒25% (Fig. 3a,b; *t*-test *P* < 3E-05) as reported previously^1^. All protection conferred by *cram-1* KD was negated, however, by concurrent knockdown of *rad-23* (using a 1:1 mixture of RNAi constructs, *t*-test *P* < E-4).

We next assessed paralysis in CL4176 [*myo-3*p::Aβ_1–42_], a model of β-amyloidosis^1,21^, using a similar dual-RNAi experimental design (Fig. 3c). A synchronous cohort of worms expressing Aβ_1–42_ in muscle was fed dsRNA that targets *rad-23, cram-1*, or both, starting 40 h after recovery of embryos. Worms were induced to express Aβ_1–42_ (by upshift to 25 °C at 48 h post-lysis), and paralysis was scored 48–52 h later. Data from 3 such experiments are presented in Fig. 3c. In all experiments, knockdown of *cram-1* was highly protective against paralysis, decreasing its incidence 2- to 3-fold (*P* < 0.001), whereas concurrent *rad-23* KD fully reversed that protection (*P* < 0.006 for reversal).

These experiments indicate that *cram-1* KD requires *rad-23* activity to rescue phenotypes resulting from aggregation of either Q40 (mimicking Huntington’s disease) or Aβ_1–42_ amyloid (observed in Alzheimer’s). CRAM-1 could perhaps compete with *ce*RAD-23 for binding to ubiquitin-tagged proteins, thereby blocking the normal role of *ce*RAD-23, i. e. delivery of misfolded proteins to 26S proteasomes for degradation. In principle, CRAM-1 protein might instead interact directly with *ce*RAD-23, with pro-aggregative effects mediated by that interaction. Our evidence does not support this alternative, however, since docking simulations do not predict a stable CRAM-1:*ce*RAD-23 interaction, and *in vivo* cross-linking studies revealed no linked peptides that indicate contact between these two proteins.

### Aggregate reduction via *cram-1* knockdown impedes ATG-7 inhibition

**Figure 4:**
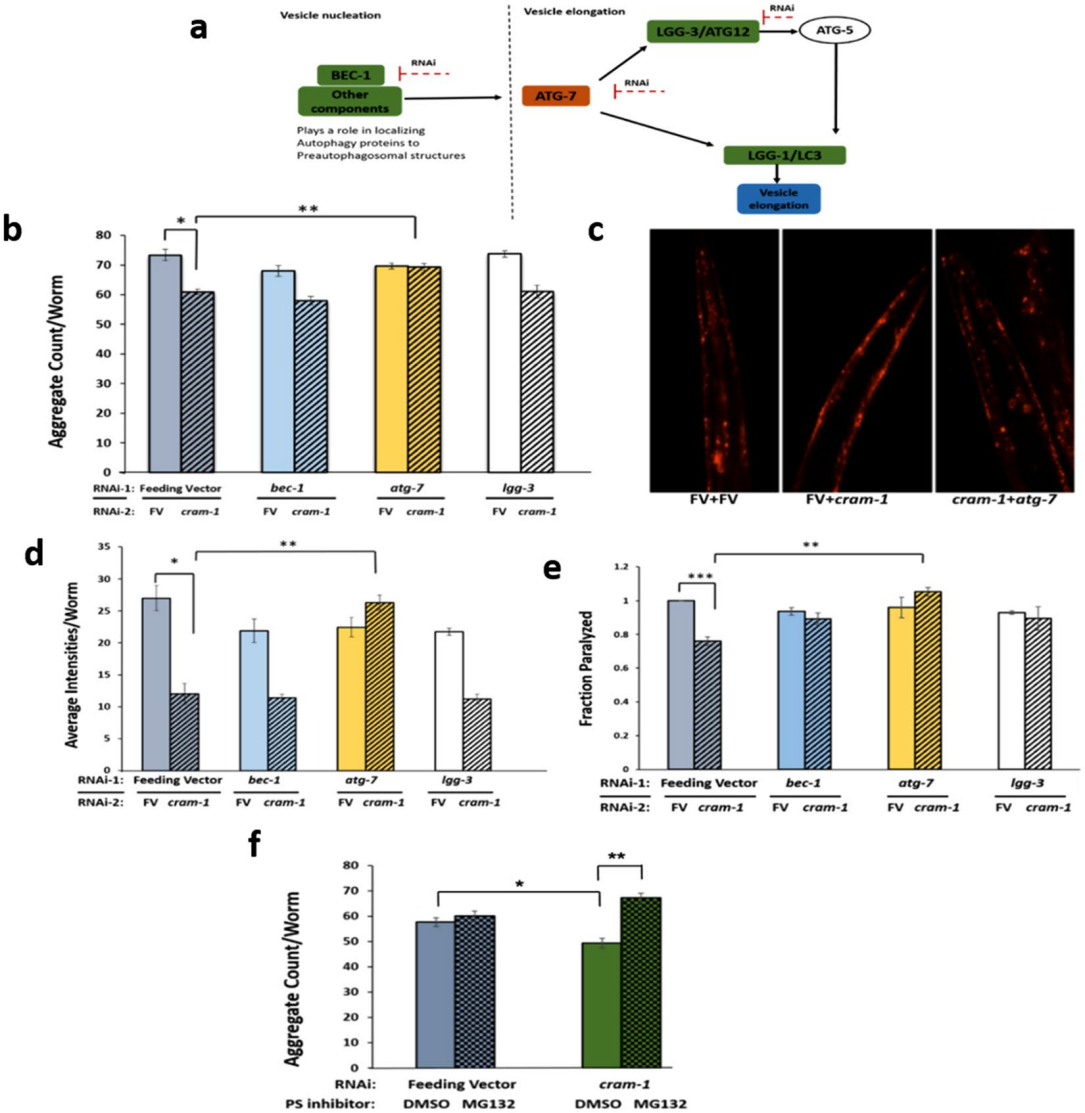
*Cram-1* knockdown reduces aggregation of Q40::YFP and formation of Aβ_1–42_ amyloid, dependent on proteasome and ATG-7 functions. (**a**) Schematic depiction of RNAi targets and their roles in autophagy pathways. (**b**) For dual RNAi, AM141 worms were fed from the L3/L4 molt on bacteria carrying empty FV, or FV expressing dsRNAs to target *lgg-3, bec-1*, or *atg-7*, each mixed 1:1 with RNAi against *cram-1* (striped bars, ± SEM) or empty FV (solid bars, ± SEM). Aggregates per worm were counted on D5_PH_. *P < 2E–05, in a 1-tailed *t*-test comparing FV to [*cram-1*_*KD*_ + FV]; **P < 0.0002, in a 2-tailed *t-*test comparing [*cram-1*_*KD*_ + FV] to [*cram-1*_*KD*_ + *atg-7*_*KD*_]. (**c**,**d**) LN149 worms expressing mCherry::ubiquitin in muscle were fed from the L3/L4 molt through D8_PH_, on dual-RNAi as described for panel b. Images of mCherry fluorescence are shown in c, and mean mCherry intensity per worm ± SEM is summarized in d. *P < 1E–04, in 1-tailed *t*-test between FV and [*cram-1*_*KD*_ + FV]; **P < 6E–05, for a 2-tailed *t*-test of [*cram-1*_*KD*_ + FV] vs. [*cram-1*_*KD*_ + *atg-7*_*KD*_]. (**e**) CL4176 worms, expressing human Aβ_1–42_ in muscle, were fed dual RNAi as in b. Paralysis, assessed 36–40 h after induction for 50–100 worms/group, is shown as mean ± SEM, treating each experiment as one data point per group. One replicate value for [*lgg-3*_*KD*_ + *cram-1*_*KD*_] was excluded as an outlier, >8.5 SDs from the mean of all other values (*P* < 6E–10). **Significance by 1-tailed paired *t*-test, *P* < 0.01. ***P < 1E–40, assuming a normal distribution. (**f**) *C. elegans* strain AM141 (*unc54p/Q40::yfp*) was fed from hatch on bacteria containing feeding vector (FV) or FV expressing dsRNA to target *cram-1*. Worms were treated from the L3/L4 molt onward with either 20-μM MG132 to inhibit proteasomes (cross-hatched bars), or vehicle only (solid bars). Aggregates per worm were counted on D5_PH_ for 10–15 worms/group. *P ≈ 0.003, 1-tailed *t*-test between FV and *cram-1*_*KD*_; **P ≈ 1E–05, 2-tailed *t*-test for [*cram-1*_*KD*_ + DMSO] vs. [*cram-1*_*KD*_ + MG132].

We next investigated the role of autophagy in *cram-1* KD-mediated protection from aggregation. Key steps in autophagy (summarized in Fig. 4a) — nucleation (via *bec-1*), and protein conjugation and vesicle elongation (via *atg-7* and *lgg-3*) — were disrupted by RNAi KD, ±RNAi to *cram-1*. Synchronized AM141 worms were transferred from bacteria harboring empty feeding vector (FV) to dual-RNAi combinations as indicated, beginning at the L3/L4 transition to minimize RNAi disruption of development^1^. Q40::YFP aggregate numbers per worm were counted at day-5 post-hatch, for ≥12 worms per group. KD of *atg-7* (encoding an E1-like ubiquitin-activating enzyme involved in autophagosome targeting) blocked the anti-aggregation effects of *cram-1* KD (yellow bars, Fig. 4b). In contrast, KD of *lgg-3* or *bec-1* did not diminish aggregation protection by *cram-1* KD relative to FV controls (Fig. 4b). These results imply that protection via *cram-1* KD (striped bars in Fig. 4) depends on ATG-7 but not on LGG-1 or BEC-1.

To visualize the “backlog” of ubiquitin-tagged substrates, we employed a UPS-reporter strain (LN149) expressing both mCherry::ubiquitin and Q82::YFP in body-wall muscle. In this model, Q82::YFP rapidly forms aggregates in adolescent or mature worms, while mCherry:: ubiquitin serves as a *pre-ubiquitinated* reporter substrate that co-localizes with Q82::YFP. When UPS degradation functions normally, mCherry::ubiquitin is efficiently cleared, whereas residual mCherry signal indicates compromise of UPS clearance. Synchronized L3/L4 worms were transferred onto dual-RNAi plates targeting candidate genes, as in the experiment described above. Mean mCherry fluorescence per worm (a measure of uncleared, ubiquitinated substrate) was quantified on day 8 post-hatch (Fig. 4c,d). *Cram-1* KD (striped bars) reduced the steady-state amount of mCherry::ubiquitin by 50–60% (each *t*-test *P* < 0.0002) whether combined with FV, *lgg-3* KD, or *bec-1* KD, indicating that neither LGG-3 nor BEC-1 is required for protection by *cram-1* KD. Dual RNAi knockdown of *atg-7* and *cram-1*, however, restored the mCherry::ubiquitin to at least the level of worms given either FV or *atg-7* RNAi alone (N.S.), well over twice the signal seen after *cram-1* KD alone [*cram-1*_KD_ + FV], *P* < 6E–05.

We next employed a nematode model of amyloidopathy, strain CL4176 [*myo-3*p::Aβ_1–42_], that expresses the human amyloid peptide Aβ_1–42_ in body-wall muscle, causing paralysis 2 days post-induction. For most experiments, as in previous studies^1^, Aβ_1–42_ synthesis was induced by upshift from 20° to 25°C at the L3/L4 larval molt (~48 h after egg isolation), and paralysis was scored in young adults ~42 h later. To minimize possible developmental disruption, RNAi against target genes (candidate autophagy genes, as above) began at the L2/L3 larval transition, 8–10 h prior to upshift, and was maintained continuously thereafter. Consistent with our previous results, RNAi targeting *cram-1* [FV + *cram-1*_KD_] reduced paralysis by 20–40%. In all 3 experiments conducted, this difference was significant at 36 and/or 48 h post-upshift (each Chi^2^ test *P* < 0.02; data not shown). Treating each of the three experiments as a single point per group, *cram-1* KD robustly reduced paralysis (*P* = 0.002 by 1-tailed paired *t*-test, Fig. 4e, gray bars).

Protection by *cram-1* KD was fully reversed by concurrent knockdown of *atg-7* and was partially blocked, although not significantly, by concurrent knockdown of *lgg-3* or *bec-1* (Fig. 4e). That is, [*cram-1*_KD_ + *atg-7*_KD_] differed significantly from [cram-1_KD_ + FV] at 36 or 48 h post-upshift within each experiment (each Chi^2^ *P* < 0.05), and for the three experiments combined (*P* < 0.01, 1-tailed paired *t*-test, Fig. 4e). Protection against *cram-1* knockdown was also fully reversed when 26 S proteasome function was inhibited by MG132 (Fig. 4f; *P* < 10^−5^). The above results imply that protection against aggregation by *cram-1* knockdown requires both ATG-7 activity and active 26S proteasomes.

### Protein unfolding increases the aggregation propensity of CRAM-1

**Figure 5:**
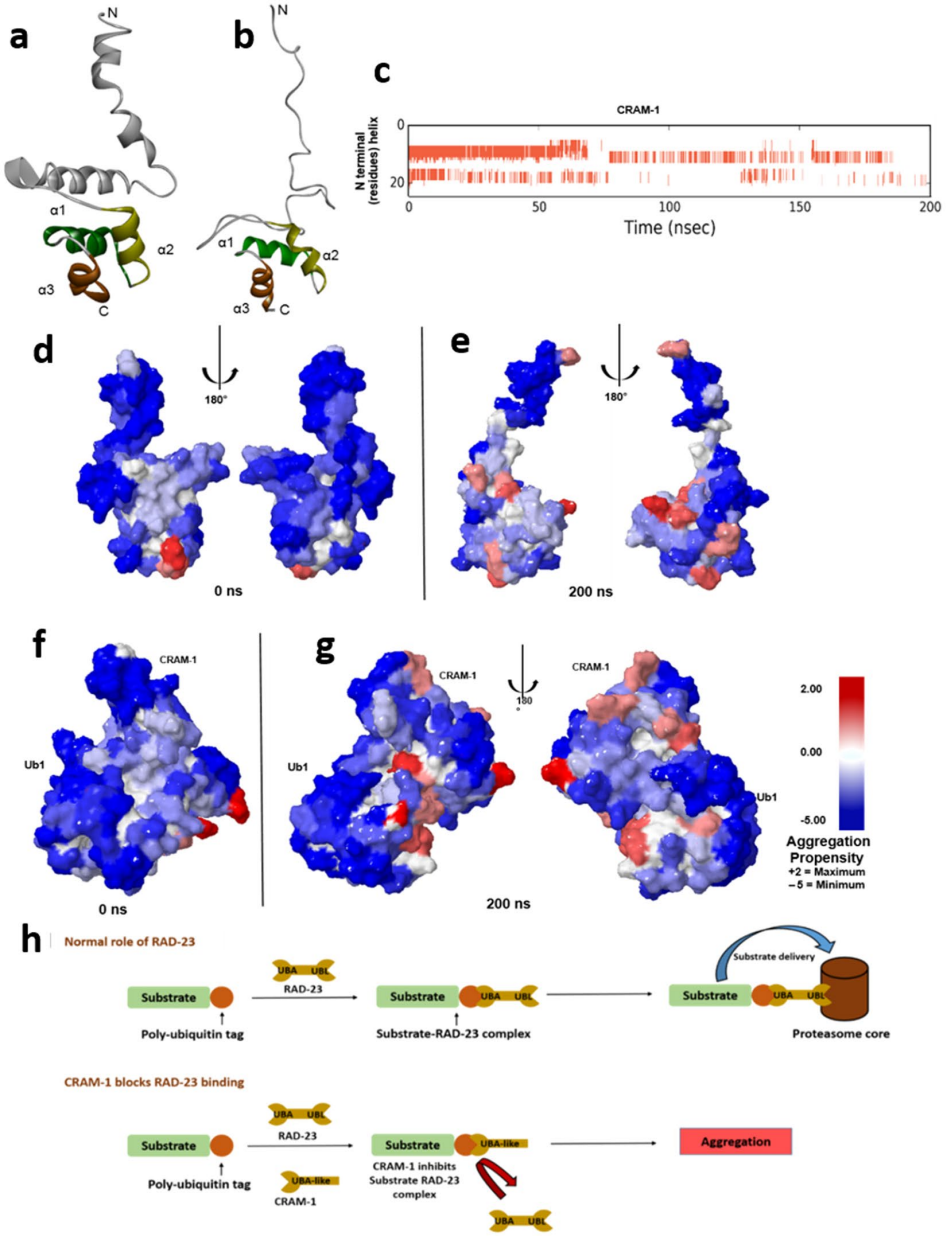
Atomistic molecular-dynamic simulation predicts aggregation-prone regions in CRAM-1 due to structural unfolding. (**a**,**b**) MD simulation in Desmond (Desmond Molecular Dynamics System, ver. 2016.4, D.E. Shaw Research, New York, NY) showing the unfolding of protein structure from initial (**a**) to simulation-stability (**b**) conformations. (**c**) Helical regions (red bars) indicate unfolding of N-terminal residues, plotted against simulation time (x axis). (**d**–**g**) Predicted aggregation propensity (see scale at right) of CRAM-1 alone before simulation of structural rearrangement (**d**), or at the end of simulation (**e**) and of CRAM-1 bound to mono-ubiquitin (ub1) before simulation (**f**), or at the end of simulation (**g**). (**h**) Schematic depiction of a proposed mechanism, wherein CRAM-1 competes with RAD-23 for binding to ubiquitin, thus impeding clearance of ubiquitinated substrates.

Because CRAM-1 was initially identified in day-7 (post-reproductive) insoluble aggregates from a *C. elegans* strain that expresses huntingtin-like polyglutamine arrays^1^, we analyzed the aggregation propensity and disordered regions of the CRAM-1 protein structure, based on its structural dynamics in atomistic molecular-dynamic simulations. We modeled full-length CRAM-1 by the same fold recognition/*ab-initio* structure prediction method we had used to model its C-terminal UBA-like region. The full model predicted the same UBA-like cluster of 3 α-helices (3 turns each) joined by loops, as predicted for the C-terminal model alone. The N-terminal region contains 2 weaker α-helices (4 turns each) connected by a loop (Fig. 5a). This full-length CRAM-1 model supersedes our previous model^1^, which was derived by homology (with <50% template identity) and iterative loop refinement.

Structural changes were documented during 200-ns simulations. Two of three independent simulations showed complete unfolding of the weak N-terminal helices (Fig. 5a–c), but stable retention of C-terminal helices comprising the UBA-like domain in all 3 simulations (Supplementary video 1). The aggregation propensities for initial and post-simulation (200-ns) conformations of CRAM-1 were calculated using AGGRESCAN3D. The results show that aggregation-prone regions become increasingly exposed with protein unfolding (Fig. 5d,e).

To more rigorously assess aggregation propensity, we performed computational docking of CRAM-1 with 1000 random proteins. CRAM-1 showed high affinity for a substantial majority of proteins: >85% of interactions fell between −150 and −260 kcal/mol, with −207 kcal/mol average interaction energy (Supplementary Fig. 1d), supporting the predicted exposure of aggregation-prone regions in CRAM-1. To determine whether this level of interaction is exceptional relative to other proteins, we simulated protein-protein interactions within each of 3 sets of 25 proteins (i.e. 75 total) taken at random from 1000 proteins used in Supplementary Fig. 1d. The number of pairwise interactions per set is *C(25,2)* = 300, or 325 including homodimers, totaling 975 interactions for 3 sets (Supplementary Fig. 1e). The average interaction energy for the 3 control sets is −96.0 ± 12.6 (SEM) kcal/mol, less than half of the CRAM-1 interaction energies (−207.3 ± 1.7 kcal/mol).

We then asked whether the aggregation propensity of CRAM-1 alters when its C-terminal UBA-like domain binds ubiquitin (Ub1) during a 200-ns atomistic simulation. The results predict stable binding of full-length CRAM-1 to ubiquitin(s), consistent with the prediction from C-terminal modeling. Remarkably, ubiquitin binding did not diminish the aggregation-propensity of any CRAM-1 regions, implying that CRAM-1 could aggregate with other proteins in addition to ubiquitin-tagged substrates (Fig. 5f,g). Based on our computational prediction and experimental data, we postulate that CRAM-1 aggregation involves both “general” interactions among disordered proteins, and annealing specific to ubiquitin-tagged proteins. This dual mechanism could account for the surprising extent of protection conferred by *cram-1* knockdown (Fig. 5h).

### SERF-2, a close human ortholog of CRAM-1, is disordered and aggregation-prone

**Figure 6:**
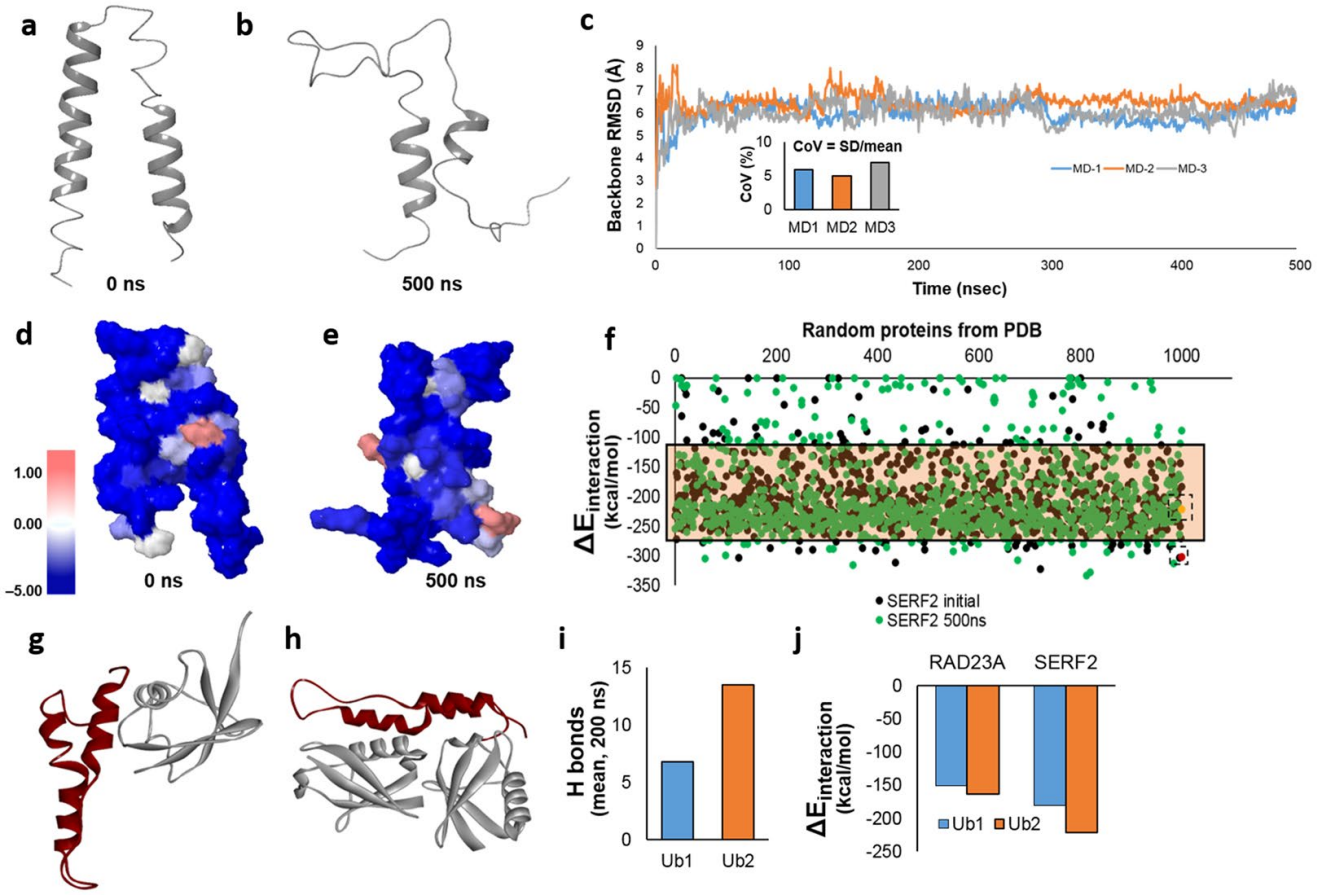
MD simulation and protein-protein interactions predict that SERF2 is aggregation-prone. (**a**,**b**) MD simulation of SERF2, in Desmond (Desmond Molecular Dynamics System, ver. 2016.4, Shaw Research, New York), indicating its initial state (**a**) and the unfolded state after 500 ns (**b**). (**c**) RMSD for three independent simulations of SERF2 structure monitored throughout the simulation. The inset shows the coefficient of variation for RMSD in each simulation. (**d**,**e**) Predicted aggregation-prone regions (see scale at left) for SERF2 in its initial conformation (**d**) and unfolded conformation after 500 ns of simulation (**e**). (**f**) Interaction energies were predicted for SERF2 in its initial (black dots) and simulated unfolded (green dots) conformations when interacting with 1000 random proteins from the PDB databank. Interaction energies are also shown for SERF2 dimer in its initial conformation (boxed red dot) and in a subsequent unfolded state (boxed yellow dot). The tinted rectangle indicates the 90% confidence interval for SERF2 interaction energies. (**g**,**h**) Predicted structures are shown for SERF2 interacting with Ub1 (**g**) or Ub2 (**h**). (**i**) Average H-bond number was calculated for a single SERF2 molecule interacting with Ub1 or Ub2, over a 200-ns simulation. (**j**) Interaction energies were predicted for RAD23A or SERF2 molecules binding to mono- and di-ubiquitin (Ub1 and Ub2, respectively).

By sequential tracking of the last common ancestral protein along the phylogenetic tree, we identified SERF2 as the closest human ortholog of CRAM-1, despite the absence of significant sequence similarity between these proteins^1^. Based on their common origin, we anticipated that SERF2 may show structural and/or functional conservation to CRAM-1 and may play a similar role in mammalian protein aggregation. *Ab initio* modelling of the SERF2 structure indicates 2 helices connected by a loop (Fig. 6a), with little conformational resemblance to CRAM-1 or any other UBA-domain-containing protein. Sequence-based algorithms^35^ indicate that 100% of SERF2 residues have high potential for disorder, and 95% are predicted to be solvent accessible. We then analyzed the structural dynamics of SERF2 by atomistic molecular-dynamic (MD) simulation for 0.5 µs. Like CRAM-1, SERF2 undergoes extensive unfolding of helices to random coils (Fig. 6b), which could cause loss of function and increased aggregation propensity.

MD simulations were performed in triplicate, allowing calculation of average SERF2 structural properties. All three simulations stabilized rapidly (<20 ns) at similar root-mean-square deviation (RMSD) values (Fig. 6c): 6.0, 6.43 and 6.05 Å (mean = 6.16 Å). These data are consistent with a preferred, relatively stable but largely unfolded conformation of SERF2, which differs substantially from its initial structure. The Coefficient of Variation (SD/mean) of RMSD beyond 35 ns was 5–7%, indicating rather little structural change after initial SERF2 unfolding. Aggregation propensities were calculated for initial and post-simulation (500-ns) SERF2 structures. Unlike CRAM-1, SERF2 did not increase its predicted aggregation propensity as it unfolded (Fig. 6e). This may reflect that, based on its high intrinsic aggregation propensity, SERF2 lacks initially-buried hydrophobic residues that could be exposed during MD simulation.

We next simulated SERF2 interactions with 1000 proteins of known structure, randomly taken from PDB (https://www.rcsb.org/). SERF2 was predicted to interact strongly with most of these proteins, in both its initial state (Fig. 6f; black dots) and in the unfolded state observed at 500 ns simulation (green dots). Of all predicted SERF2 interactions, 90% fall between −111 and −267 kcal/mol (shaded rectangle in Fig. 6f, Supplementary Fig. 1e), whereas only 34% of 1000 random protein-protein interactions fall within this range (average ΔE −96.0 kcal/mol), consistent with SERF2 being far more interaction-prone than most proteins.

We then asked whether SERF2 and CRAM-1, despite the absence of structural similarity, show functional conservation regarding interaction with ubiquitin. Protein-protein docking predicts SERF2 interaction with mono- or di-ubiquitin (Ub1, Ub2), with interaction energies even more favorable than those for RAD23A (Fig. 6g–j). We note that the ubiquitin facets that interact with SERF2 differ from those contacted by CRAM-1 or any UBA-domain protein including MUD1 or *hs*RAD23A (human). MD simulations of protein-protein complexes indicate stable binding of SERF2 to ubiquitin (Ub1 & Ub2). Moreover, the number of hydrogen bonds between SERF2 and ubiquitin is stable throughout each simulation, averaging 6.7 H bonds per ubiquitin moiety (Fig. 6i).

Since the binding energy of SERF2 to ubiquitin exceeds that of RAD23A (Fig. 6j), we expected that SERF2 binding to ubiquitin would block RAD23A-ubiquitin interaction. Analysis of a simulated 3-molecule interaction, comprising RAD23A, ubiquitin, and SERF2, supports this prediction (Supplementary Fig. 1f). In additional simulations, SERF2 spontaneously formed homodimers with an interaction energy (ΔE_interaction_) of −302.9 kcal/mol (Fig. 6f, red dot), lying at the 0.4^th^ percentile for simulated interactions of SERF2 with 1000 randomly chosen proteins. These data argue that SERF2 could promote aggregate progression, perhaps by the mechanisms inferred for CRAM-1: obstructing RAD23-ubiquitin interaction, and propensity to aggregation in general.

### *SERF2* knockdown reduces amyloid aggregation in human neuroblastoma cells

**Figure 7:**
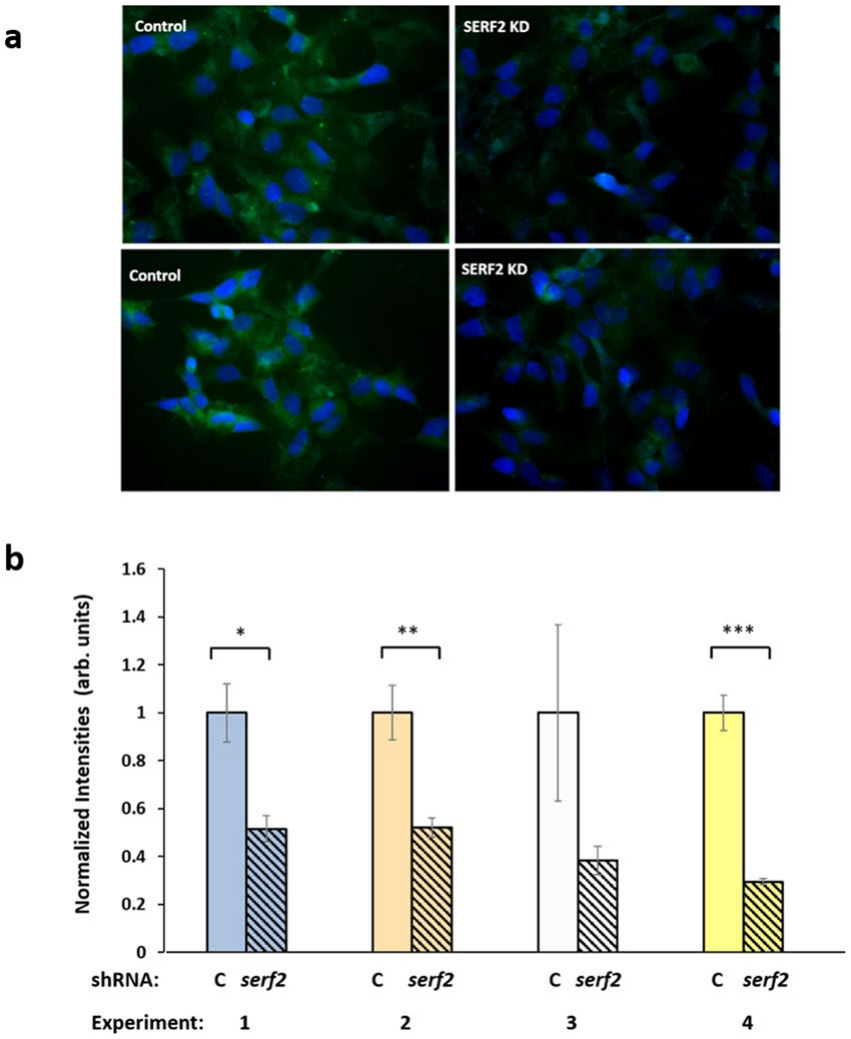
*SERF2* knockdown protects against amyloid aggregation in human neural cells. (**a**) SH-SY5Y-APP_Sw_ neuroblastoma cells were transfected with *SERF2* shRNA, and stained with thioflavin T at 48 h post-transfection. Amyloid fluorescence is displayed in green, and nuclei (counterstained with DAPI) appear blue. (**b**) Thioflavin-T fluorescence was quantified and divided by the number of DAPI-stained nuclei per field, to estimate amyloid deposition per cell. In 4 independent experiments, *SERF-2* knockdown reduced amyloid by 50–70%, attaining statistical significance in 3 of 4 experiments. Fluorescence intensity per cell is plotted ± SEM, normalized to the control mean for each experiment. *P < 0.01 by 2-tailed *t-*test (initial experiment); **P < 0.01 by 1-tailed *t-*test; ***P < 0.001 by 1-tailed *t-*test.

Computational studies indicate that SERF2 is disordered, aggregation-prone, and able to interact with damaged or misfolded proteins via their ubiquitin tags, or directly with disordered regions. Knockdown of CRAM-1, the *C. elegans* ortholog of SERF2, reduces aggregation and associated toxicity in *C. elegans* models of protein aggregation associated with neurodegenerative diseases^1^ (see also Figs 1–5). We therefore used short-hairpin RNA (shRNA) constructs to knock down SERF2 expression in a human neuroblastoma cell line expressing an aggregation-prone mutation of amyloid precursor protein (SY5Y-APP_Sw_). These cells produce an overabundance of Aβ_1–42_ and continuously form extracellular amyloid aggregates. Cells were transfected with shRNA against *Serf2* or *Serf1a*, after which cells were maintained 48 h at 37 °C and then stained for amyloid. Transfected cells, and non-transfected control cells, were incubated in 0.1% (w/v) thioflavin T followed by DAPI (see Methods) and imaged for amyloid fluorescence (thioflavin: amyloid excitation peak at 450 nm; emission at 482 nm) and for DNA (DAPI: DNA excitation at 358 nm; emission at 461 nm) as shown in Fig. 7a. ShRNA knockdown of SERF2 consistently reduced amyloid aggregation by 50–70% in four independent experiments (Fig. 7a,b), three of which showed significant shifts. Parallel experiments targeting SERF1A, a somewhat more distant ortholog of CRAM-1^1^, also reduced amyloid aggregation by ~2-fold (2-tailed *t*-test *P* < 6E–07) in a single experiment (Supplementary Fig. 1g). These data corroborate our computational predictions that SERF2 could play a vital role in aggregate progression.

## Discussion

We identified key properties of the *C. elegans* CRAM-1 protein, and its closest human ortholog, SERF2, which are critical in promoting aggregation. CRAM-1 and SERF2 are predominantly-disordered proteins containing aggregation-prone regions^1^. The CRAM-1 C-terminal region (residues 54–96) comprises 3 moderately stable α-helices connected by loops, in a conformation that closely matches the *ce*RAD-23 UBA-domain structure^28^ (Fig. 1c,d). UBA domains primarily bind ubiquitin^28,30^, with increasing affinity as ubiquitin chains grow, favoring specific conveyance of poly-ubiquitinated substrates to the proteasome^24,31^. CRAM-1, despite lacking sequence similarity to known UBA domains, has striking *structural* homology to *ce*RAD-23 (RMSD = 1.02 Å) and its human ortholog, *hs*RAD23A (RMSD = 1.14 Å), suggesting that it may bind poly-ubiquitin.

We previously identified CRAM-1 as a *C. elegans* protein present in sarcosyl-insoluble protein aggregates and strongly favoring polyglutamine-seeded protein aggregation. Molecular modeling and atomistic dynamic simulations indicate its propensity to bind ubiquitin(s) via the UBA-like domain, interacting with the same residues of ubiquitin that *ce*RAD-23 binds. This interaction could prevent *ce*RAD-23 interaction with ubiquitin, which is a critical step in cargo conveyance to 26S proteasomes. In multiple nematode models of neurodegeneration-like protein aggregation, RNAi knockdown of *cram-1* protected against aggregation and associated traits^1^ — protection that is lost if UPS is inhibited either by a proteasome-inactivating drug, MG132 (Fig. 3a), or by RNAi targeting *pas-4*, which encodes an essential α subunit of the 20S proteasome^1^.

We propose several routes through which CRAM-1 could impede protein aggregation. RAD23A interacts with poly-ubiquitin chains through its UBA domain, and delivers cargo to proteasomes by docking its UBL domain to the RPN10 proteasome regulatory subunit. A deletion mutant of RAD23A, lacking a UBL domain, causes a severe protein-degradation deficiency^15,17,36^, conjectured to involve a “decoy” mechanism in which truncated RAD23A competes with intact RAD23A for protein cargos to be degraded. On the other hand, RAD23A overexpression also blocks ubiquitin chain elongation, resulting in poor UPS clearance^37^; and likewise, a UBA domain termed p47-RAT inhibits chain elongation upon ubiquitin binding^38^. CRAM-1 could compromise proteostasis by either of these mechanisms: competition with a UBA-domain-containing shuttle protein, such as *ce*RAD-23, for binding to poly-ubiquitin, or binding to “immature” mono- and oligo-ubiquitin chains and disrupting their elongation. Both mechanisms are consistent with our *in vivo* results indicating that *cram-1* KD relieves protein aggregation, but only the decoy model unambiguously predicts that *ce*RAD-23 KD would negate all beneficial effects of simultaneous CRAM-1 KD. Previous *C. elegans* studies had shown that Q40::YFP aggregates are processed chiefly by the UPS system^1,39^.

Autophagy is another critical protein-degradation pathway important for handling bulky cargos, including protein aggregates and damaged mitochondria^40–42^. We assessed several genes that play essential roles in autophagy pathways. ATG7 (an E1-like ubiquitin-activating enzyme) is a key player in autophagy; RNAi against *atg-7* was reported to block autophagy in *C. elegans*, and thereby increase sequestosome1/p62 levels, which in turn impairs 26S proteasomes by exceeding their capacity^43^. *Cram-1* knockdown relieves protein aggregation, but this rescue vanishes when autophagy is disrupted by *atg-7* KD, whereas RNAi targeting *lgg-3* and *bec-1* appeared to be less effective (Fig. 4e). These results led us to postulate that, in dual KDs targeting *atg-7* and *cram-1*, impairment of autophagy by *atg-7* RNAi indirectly suppresses UPS as well, preventing any benefit of *cram-1* knockdown. Like siRNA targeting just *rad-23* (Fig. 3b), knockdown of *atg-7* alone (Fig. 4b–e) did not elevate aggregation relative to controls — in reporter strains AM141 (*Q40::yfp*), LN149 (*ubq::mCherry, Q82::yfp*), or CL4176 (*Aβ*_*1–42*_) — but nevertheless fully blocked the protective effects of *cram-1* knockdown.

Molecular-dynamic simulations of either CRAM-1 alone, or CRAM-1 in complex with ubiquitin(s), identified structural fluctuations in the N-terminal α-helical domains of CRAM-1, increasing the exposure of aggregation-prone regions (Fig. 5). Comparing aggregation propensities of initial and MD-simulation conformations of CRAM-1 alone (Fig. 5d,e), vs. CRAM-1 complexed with ubiquitin (Fig. 5f,g), indicates that aggregation-prone regions are exposed by unfolding of the CRAM-1. *In silico* modeling of interactions with random proteins implies that CRAM-1 could stably bind many other proteins (Supplementary Fig. 1d).

The above results suggest an explanation for the surprisingly large effect of CRAM-1 on the aggregate burden. In addition to acting as a decoy competing for ubiquitin sites with UPS shuttle proteins, and perhaps compromising ubiquitin chain elongation, the ubiquitin-bound form of CRAM-1 unveils its own aggregation-inclined aspects and also those of its binding partner (ubiquitin in Fig. 5g), enhancing the likelihood that both will adhere to a growing aggregate.

An RNAi screen for modifiers of protein aggregation in *C. elegans* identified only one gene, *moag-4* (the nematode ortholog of human *SERF1*), for which knockdown protected nematodes against Q40::YFP aggregation^44,45^. Knockdown of *moag-4* was thought to confer protection by mechanisms independent of UPS or autophagy, but involving direct interaction with amyloid^44^. CRAM-1 retains no sequence similarity to SERF2, indicating that their genes diverged long ago, and/or experienced very little selective pressure for protein-sequence conservation; likewise, CRAM-1 shows no homology to MOAG-4 or SERF1.

SERF2 (small EDRK-rich factor 2) is a small (59 residue), ubiquitously-expressed protein rich in basic residues lysine (~20%) and arginine (11%). Structural and dynamic modeling of SERF2 predicted two α-helices linked by a short random coil, which departs from the typical UBA-domain bundle of three α-helices but instead resembles the helix-turn-helix structure of the ubiquitin binding motif (UBM)^46^. Despite their differences, both SERF2 and CRAM-1 are predicted to interact stably with ubiquitin chains, and KDs of both protect against aggregation. In SY5Y-APP_Sw_ neuroblastoma cells that form extracellular amyloid, SERF2 knockdown reduced those aggregates by 50–70%. While SERF2 may employ a decoy mechanism as proposed for CRAM-1, it is also predicted to form stable interactions with many partners (Fig. 6f; Supplementary Fig. 1e), conferring the potential to form branch points critical to aggregate growth. We note that multiple protein interactions were recently reported for SERF2 in a yeast two-hybrid system, including the PA28γ subunit of a 20S proteasome-activating complex, and several RNA-binding and RNA-processing proteins^47^.

Based on i*n silico* interaction with randomly selected proteins, we predict that SERF2 could interact with many (perhaps most) proteins. Together, these findings suggest that SERF2 is exceptionally interactive, due to its unstructured and flexible nature — properties that are consistent with roles in aggregate progression and (through ubiquitin binding) disruption of aggregate-clearance pathways. Further studies assessing the mechanistic role of SERF2 in aggregate accrual should shed light on the balance between aggregate formation and clearance, while at the same time suggesting new therapeutic targets for the many age-progressive diseases that feature protein aggregation.

## Methods

### Strains and maintenance

*C. elegans* strains AM141 [*unc-54p::Q40::yfp*] and CL4176 [*smg-1*^*ts*^; *myo-3p::Aβ*_*1–42*_*::let-851* [*3′-UTR*]; *rol-6(su1006)*] were obtained from the Caenorhabditis Genetics Center (CGC). The LN149 strain [*unc-54p::Q82::yfp*; *unc-54p:: mCherry::ubiquitin*] was kindly provided by Lynn Boyd and Gregory Skibinski (Univ. Alabama, Huntsville, AL).

The strains listed above were routinely maintained on regular solid-agar nematode growth medium (NGM) seeded with *E. coli* (strain OP50) bacteria, at 20 °C except for CL4176 paralysis experiments in which worms were induced by upshift from 20° to 25° C at the L3/L4 transition (~48 hours after eggs hatched). For knockdown experiments, well-fed, gravid day-1 adults were lysed in alkaline hypochlorite to release unlaid eggs, which were allowed to hatch on plates with *E. coli* (strain HT115) RNAi sublines, each carrying a plasmid expressing an RNAi construct to target a gene of interest^48^. If RNAi exposures caused developmental delays or defects, eggs were instead hatched on HT115 bacteria carrying empty plasmid vector, and transferred only at the L3/L4 molt to RNAi-expressing bacteria, on which they were subsequently maintained. Dual-RNAi experiments used 1:1 mixtures of HT115 bacteria carrying two distinct RNAi-expressing plasmids, or a subline carrying an RNAi-expressing plasmid mixed 1:1 with empty-vector control bacteria.

### Visualization of reporter strains

AM141 worms were imaged on a Nikon Eclipse E600 fluorescence microscope fitted with a Nikon CoolSnap ES camera, and Q40::YFP aggregates were counted (DotCount, http://reuter.mit.edu/software/dotcount/). Incident and emitted light were filtered to 490 ± 20 nm and 535 ± 30 nm, respectively. Similarly, red and yellow fluorescence were imaged in LN149 adults (expressing *unc54p/mCherry::ubiquitin* and *unc54p/Q82::yfp* in body wall muscle), and the average fluorescence intensity of mCherry::ubiquitin foci was quantified with FIJI (ImageJ).

### Paralysis assay

The CL4176 strain, expressing Aβ_1–42_ in muscle, was synchronized by lysis as above, and eggs were transferred onto 60-mm agar plates seeded with bacteria expressing dsRNAs against targeted genes (or to plates seeded with dual dsRNA vectors in 1:1 ratio). Worms in experimental groups were upshifted from 20° to 25° C at the L3/L4 transition (47–49 h post-lysis) to induce expression of Aβ_1–42_. Paralysis of worms (defined as loss of motility in response to touch) was scored from 18-h post-upshift until motility fell below 60% in the FV (control) group. To slow development of progeny in synchronized populations, 5-fluoro-2′-deoxyuridine (FUdR) was added at a final concentration of 2 μM to RNAi plates and to control (FV) plates, each containing worms from pre-gravid (L4/adult molt, day 2.5 post-hatch) through post-gravid ages (beyond 6–7 days post-hatch).

### Structure generation

Three-dimensional structures of full length CRAM-1 and its UBA-like domain, and of *ce*RAD23, were modelled using the I-TASSER server, which performs fold recognition followed by *ab initio* prediction of structure^49^. Structures retrieved from the Protein DataBase (PDB; https://www.rcsb.org/) were human RAD23A (1F4I; C-terminal UBA(2) domain), MUD1 (1Z96), di-ubiquitin open conformer (2PE9), plus 1000 randomly chosen proteins. The full-length structure of CRAM-1 was simulated briefly (10 ns) in implicit solvent (GBSA) using the GROMACS simulation package^50^, prior to docking studies.

### Protein-protein docking

To model protein-protein interactions, the HEX 6.1 program^1,51^ was used with default parameters. Interactions from each run were ranked by ΔE_interaction_ energies, and the lowest-energy (most stable) models were chosen for dynamic simulations. Automated Linux shell scripts were written to automate large-scale computational docking studies in Hex.

### Molecular-dynamic simulation

Atomistic molecular dynamics of individual proteins and complexes were simulated using Desmond software (Desmond Molecular Dynamics System, version 2016.4, D.E. Shaw Research, New York, NY). Proteins were initially immersed in an orthorhombic box containing SPC water, pH neutralized, and salt set to 0.15-M NaCl. For MD runs, the ensemble class was set to NPT, and temperature and pressure were set to 300 °K (Nose-Hoover chain method) and 1.013 bar (Martyna-Tobias-Klein method) respectively. Velocities were randomized every 25 ps to minimize sampling bias. Each MD run was performed for 200–500 ns in duplicate or triplicate, and trajectories were analyzed using built-in packages from Desmond-Maestro, VMD and Discovery Studio (BIOVIA Discovery Studio [Ver. 17.2.0], Dassault Systèmes, San Diego [2017]).

### Cell culture and maintenance

SH-SY5Y-APP_Sw_ cells, overexpressing an aggregation-prone familial-AD mutation of amyloid precursor protein, APP_Sw_, were kindly provided by Dr. Steven Barger. These cells model Alzheimer’s-like amyloid formation to study the contribution of SERF2 to amyloidopathy. Cells were maintained at 37 °C with 5% CO_2_ in a tissue-culture incubator, in culture dishes containing DMEM-F12 (1:1) nutrient mixture (Ham’s medium) with 10% v/v fetal bovine serum.

### Lipofection and thioflavin-T assay

To knock down expression of SERF1A or SERF2, target-specific shRNAs were introduced into SH-SY5Y-APP_Sw_ cells by lipofection. Well-maintained cells were grown to confluence, then trypsinized and subcultured in 12-well plates (at 15,000–20,000 cells/well) containing antibiotic-free DMEM medium. After 24 h, shRNA against each candidate gene was introduced with Lipofectamine 2000 (Invitrogen). Transfected cells, along with control cells (mock treatment), were maintained at 37 °C for 48 hours and then incubated with 0.1% (w/v) thioflavin T in phosphate-buffered saline to stain amyloid aggregates. Total aggregate fluorescence was quantified from images using FIJI (ImageJ), with background subtraction at a rolling-ball radius of 50 for all images. To obtain the average thioflavin T fluorescence per cell, the total aggregate fluorescence in each captured image was divided by the number of cells (nuclei stained with DAPI) counted in the same image.

### Western-blot detection of CRAM-1/ubiquitin binding

Wild type N2 (Bristol) worms were fed with regular *E. coli* (OP50) bacteria and maintained at 20 °C. Adult day-1 (D1) and day-5 (D5) worms were flash frozen and homogenized. Lysates were then incubated with either biotin-tagged antibody to CRAM-1 (captured on streptavidin-coated magnetic beads), or magnetic beads coated with antibody to ubiquitin. Eluted proteins were resuspended in 2x Laemmli buffer with β-mercaptoethanol at 95 °C, and equal worm equivalents loaded on a 1% SDS, 10% (w/v) polyacrylamide gel, electrophoresed, and transferred to nylon membranes. Blots were incubated with murine antibodies to ubiquitin (Abcam) or rabbit antibodies to CRAM-1 (Genscript). Membranes were imaged using FluorChem Q (Cell Biosciences) after incubation with horseradish peroxidase (HRP)-coupled antibody to either mouse IgG or rabbit IgG.

### Statistical tests

Groups are generally compared by 2-tailed Behrens-Fisher *t* tests (conservative *t* tests for samples of unequal or unknown variances), unless the direction of the difference is known or strongly predicted, in which case a 1-tailed *t* test is used. Differences in proportions are assessed by Chi-squared (Chi^2^) or Fisher Exact tests. Control samples used for normalization across multiple experiments (e.g., bars at left of Fig. 3c) cannot be evaluated by *t* tests due to zero variance of normalized control values. The null hypothesis is instead tested by calculating the area under a normal-distribution tail for control values ≥1.00, given a normal distribution with the observed mean and SEM (0.47 ± 0.015 in Fig. 3c, for [*cram-1*_KD_ + FV]).

## Electronic supplementary material

Supplementary Figure 1

Supplementary video 1

Supplementary video 2
