# Supplementary material for: Structural insights into pro-aggregation effects of C. elegans CRAM-1 and its human ortholog SERF2
Source: Sci Rep. 2018 Oct 5;8:14891. doi: 10.1038/s41598-018-33143-1 (PMC6173753; doi:10.1038/s41598-018-33143-1)
Supplement: Supplementary file 1 — Supplementary Figure 1 [file 41598_2018_33143_MOESM1_ESM.pdf]

Structural insights into pro-aggregation effects of *C. elegans* CRAM-1 and its human ortholog SERF2

Meenakshisundaram Balasubramaniam, Srinivas Ayyadevara, Robert J. Shmookler Reis

Supplementary Figure 1

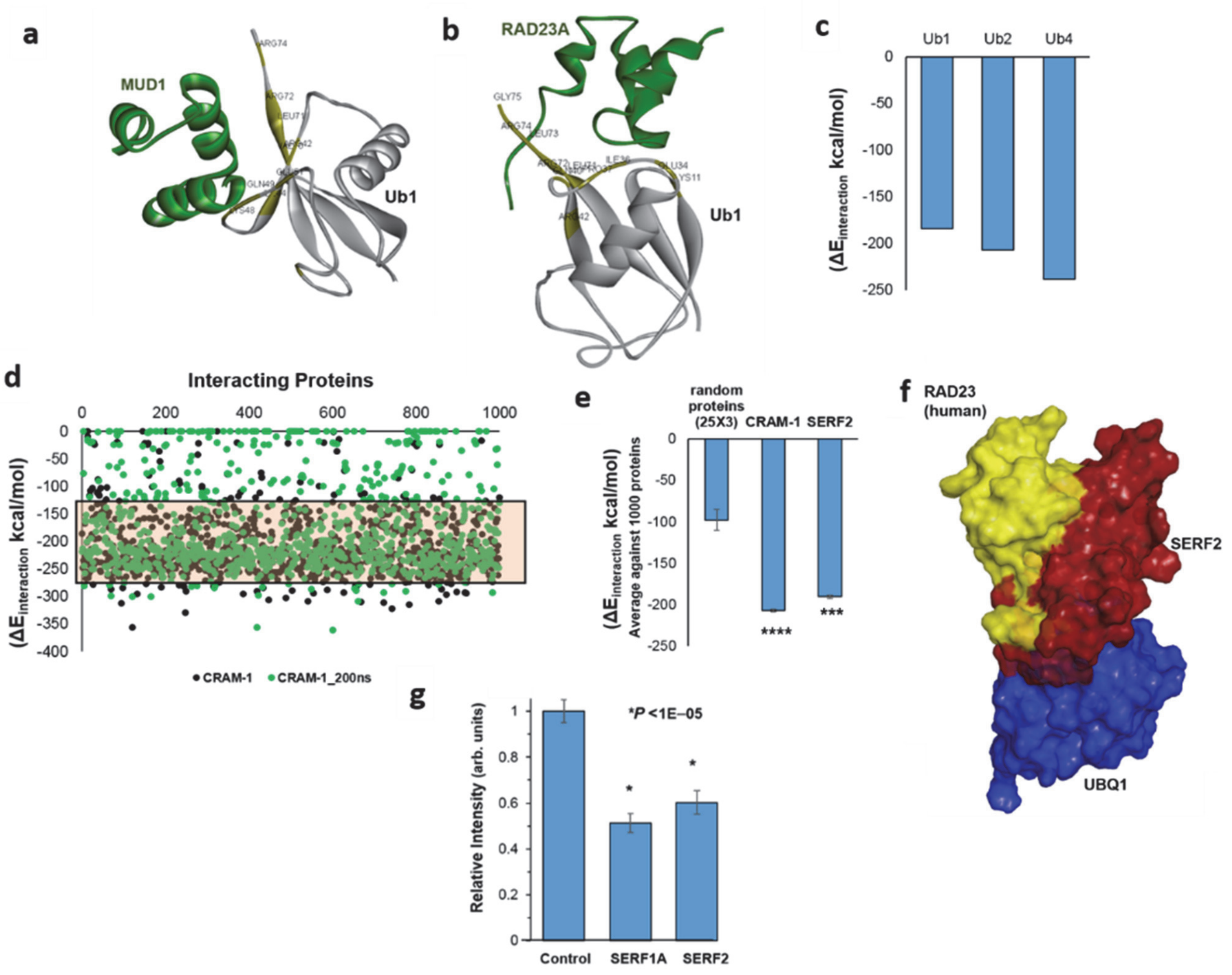

Supplementary Figure 1: (**a**) Predicted interactions of MUD1 (green) with mono-ubiquitin (gray), indicating contact residues in ubiquitin. (**b**) Predicted interactions of human RAD23A with mono-ubiquitin, indicating its contact residues. These results (**a**, **b**) agree with NMR chemical-shift assays[23, 27, 29]. (**c**) Predicted interaction energies of full length CRAM-1 interacting with mono-, di-, and tetra-ubiquitin. (**d**) Predicted interactions of CRAM-1 with 1000 random proteins. The tinted rectangle indicates the > 85% confidence interval for CRAM-1 interaction energies. (**e**) Interaction energies for random (control) protein pairings (3 sets of 25 proteins, each taken 2 at a time), or CRAM-1 or SERF2, each paired with 1000 proteins taken at random from PDB significance by 2-tailed t-test: ****P*<1E–139; *****P*<4E–193). (**f**) Predicted interactions (or absence thereof) of RAD23A (yellow) with mono-ubiquitin (blue) bound to SERF2 (red). (**g**) SH-SY5Y-APP_{Sw} neuroblastoma cells were transfected with either *SERF1A* or *SERF2* shRNA and stained with thioflavin T at 48 h post-transfection. Thioflavin-T fluorescence was quantified and divided by the number of DAPI-stained nuclei per field, to estimate amyloid deposition per cell. **P*<1E-05 by 2-tailed *t* tests.

Structural insights into pro-aggregation effects of *C. elegans* CRAM-1

and its human ortholog SERF2

Meenakshisundaram Balasubramaniam, Srinivas Ayyadevara, Robert J. Shmookler Reis

Supplementary Video 1

Supplementary video 1: Molecular-dynamic simulation of CRAM-1, over 200 ns.

Structural insights into pro-aggregation effects of *C. elegans* CRAM-1

and its human ortholog SERF2

Meenakshisundaram Balasubramaniam, Srinivas Ayyadevara, Robert J. Shmookler Reis

Supplementary Video 2

Supplementary video 2: Molecular-dynamic simulation of SERF2, over 500 ns.
